# The Role of the Plasminogen/Plasmin System in Inflammation of the Oral Cavity

**DOI:** 10.3390/cells12030445

**Published:** 2023-01-30

**Authors:** Tetiana Yatsenko, Maksym Skrypnyk, Olga Troyanovska, Morikuni Tobita, Taro Osada, Satoshi Takahashi, Koichi Hattori, Beate Heissig

**Affiliations:** 1 Department of Research Support Utilizing Bioresource Bank, Graduate School of Medicine, Juntendo University School of Medicine, 2-1-1 Hongo, Bunkyo-Ku, Tokyo 113-8421, Japan; 2 Department of Oral and Maxillofacial Surgery, Juntendo University School of Medicine, 2-1-1 Hongo, Bunkyo-Ku, Tokyo 113-8421, Japan; 3 Department of Gastroenterology, Juntendo University Urayasu Hospital, 2-1-1 Tomioka, Urayasu-Shi 279-0021, Japan; 4 Division of Clinical Genome Research, The Institute of Medical Science, The University of Tokyo, 4-6-1 Shirokanedai, Minato-Ku, Tokyo 108-8639, Japan; 5 Center for Genome and Regenerative Medicine, Graduate School of Medicine, Juntendo University, 2-1-1 Hongo, Bunkyo-Ku, Tokyo 113-8421, Japan

**Keywords:** plasmin, COVID-19, suPAR, oral cavity, salivary gland, tPA, fibrinogen, fibrinolysis, cytokine, inflammation, SARS-CoV-2

## Abstract

The oral cavity is a unique environment that consists of teeth surrounded by periodontal tissues, oral mucosae with minor salivary glands, and terminal parts of major salivary glands that open into the oral cavity. The cavity is constantly exposed to viral and microbial pathogens. Recent studies indicate that components of the plasminogen (Plg)/plasmin (Pm) system are expressed in tissues of the oral cavity, such as the salivary gland, and contribute to microbial infection and inflammation, such as periodontitis. The Plg/Pm system fulfills two major functions: (a) the destruction of fibrin deposits in the bloodstream or damaged tissues, a process called fibrinolysis, and (b) non-fibrinolytic actions that include the proteolytic modulation of proteins. One can observe both functions during inflammation. The virus that causes the coronavirus disease 2019 (COVID-19) exploits the fibrinolytic and non-fibrinolytic functions of the Plg/Pm system in the oral cavity. During COVID-19, well-established coagulopathy with the development of microthrombi requires constant activation of the fibrinolytic function. Furthermore, viral entry is modulated by receptors such as TMPRSS2, which is necessary in the oral cavity, leading to a derailed immune response that peaks in cytokine storm syndrome. This paper outlines the significance of the Plg/Pm system for infectious and inflammatory diseases that start in the oral cavity.

## 1. Introduction

**Figure 1:**
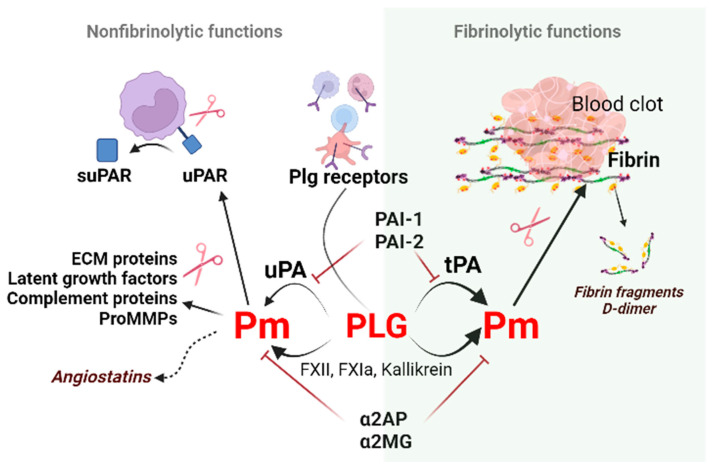
**Non-fibrinolytic and fibrinolytic functions of the plasminogen/plasmin system**. Binding and subsequent plasminogen activation via plasminogen receptors (Plg-RKT, annexin 2, actin, etc.) generate plasmin. Plasmin induces proteolytic activity on the cell surface to cleave the extracellular matrix molecules necessary for cell migration. Plasmin generated by tPA on polymer fibrin, uPA, or clotting factors on activated cell surfaces dissolves fibrin and produces fibrin fragments such as D-dimers. This process is called fibrinolysis. The non-fibrinolytic functions of plasmin include proteolytic activity towards latent growth factors, the complement component C5, and pro-MMPs resulting in changes in molecule-linked signaling pathways. The cleavage of plasmin, elastase, and MMPs generate angiostatins–kringle-containing plasminogen fragments possessing anti-angiogenic properties. Abbreviations: Pm, plasmin; tPA, tissue-type plasminogen activator; PAI-1, plasminogen activator inhibitor-1; ECM, extracellular matrix; MMPs, matrix metalloproteinases; suPAR, soluble urokinase plasminogen activator receptor; α2AP, α2-antiplasmin; α2MG, α2-macroglobulin.

The primary function of the plasminogen (Plg/Pm) system is to ensure the destruction of fibrin deposits and maintain hemostatic balance. Hemostasis is the process of blood clot formation at the site of vessel injury. There is a careful balance between thrombin-stimulated clot formation and plasmin-induced clot lysis. Abnormal bleeding occurs when there is insufficient clot formation due to decreased thrombin (e.g., from F VIII deficiency) or increased clot lysis. Conversely, non-physiological thrombosis or functional clotting occurs when excessive thrombin production is present. During the initial phase of hemostasis after tissue damage, endothelial injury and platelet plugs form a clot within 10–20 s. The sequential activation of coagulation factors (XIII-II) generates an initial hemostatic plug in 1–3 min. Fibrin, produced at the end of the coagulation cascade, adds to the clot stability by 5–10 min. Since a clot should only obstruct the vessel for a particular time, the body switches from clotting to antithrombotic control mechanisms. The activation of plasmin that leads to fibrin degradation and the occurrence of fibrin degradation fragments, a process called fibrinolysis, ultimately removes the clot to ensure tissue blood reperfusion (Figure 1).

Besides the fibrinolytic function, the Plg/Pm system can alter cell receptors, extracellular matrix molecules, or growth factors due to its proteolytic capacity. In addition, components of the Plg/Pm system participate in intracellular signaling processes, thus regulating tissue development and remodeling during wound healing, inflammation, trophoblast invasion, angiogenesis, tumor growth, etc. [1,2,3,4,5]. These functions are referred to as non-fibrinolytic functions (Figure 1).

There are two major plasminogen activators (PAs) that can activate Plg in humans: tissue-type plasminogen activator (tPA) and urokinase plasminogen activator (uPA). Both PAs are serine proteinases of the trypsin type. However, differences in their structure cause functional differences: tPA mainly converts Plg to plasmin on fibrin clots, while the activity of uPA is regulated by its interaction with the cell surface [6,7]. Plg activation is involved in tissue remodeling and inflammatory response (reviewed in [8,9]). Plg levels are high at the inflammation site and regulate the wound-healing process by activating the early inflammatory reaction [10] by increasing inflammatory cell infiltration.

“Health comes first, and it enters through the mouth”. The mouth, in academic terms, is referred to as the oral cavity [8]. The oral cavity is a structure of tissues and organs with complex organization and interaction. Initial parts of the digestive system, the immune system (Waldeyer’s tonsillar ring), and sensory-specific and non-specific receptors (taste, temperature, pain, tactile sensation, etc.) are located in the oral cavity. Both the nasal cavity and nasopharynx, as the beginning of the respiratory system, and the oral cavity are the entrance gateway for bacterial and viral infections. The first signs and symptoms of general infectious and non-infectious diseases can occur in the oral cavity (loss of taste or hemorrhagic rash on soft palatine mucosae in COVID-19, Koplik’s sign in measles, oral dryness and the decaying of multiple teeth in diabetes mellitus, etc.). The oral cavity environment is very sensitive to systemic and local changes in the organism and reacts to pathogen exposure with quantitative and qualitative changes in saliva and crevicular fluid. The Plg/Pm system of the oral fluid plays an integral part in the physiological regeneration and protective mechanisms of oral mucosae, the pathogenesis of several inflammatory or autoimmune diseases, and tumor growth in the oral cavity [11].

## 2. Regulators of Fibrinolysis

The Plg/Pm system includes the proteolytic enzyme plasmin and its inactive precursor Plg, its activators (tPA and uPA), plasmin inhibitors (α2-antiplasmin and α2-macroglobulin), and inhibitors of plasminogen activators (PAI-1 and PAI-2) [12]. These components regulate and interact with each other as well as with clotting system components, cell receptors, and pericellular adhesion molecules (Figure 1) [5]. In addition, many proteins can modulate the activity of the fibrinolytic system components: proteins such as vitronectin, thrombospondin, tetranectin, and histidine-rich glycoprotein, which can bind to Plg/Pm, fibrinolysis activators, or inhibitors.

PAs and components of the clotting cascade mediate Plg conversion to active plasmin. The tissue activator generates plasmin on fibrin and cell surface. Activation of Plg by uPA occurs on the cell surface in blood circulation or outside of it [6,12]. Like thrombin, plasmin activates protease-activated receptor (PAR)1 and PAR2, modulating platelet activation, the release of proinflammatory cytokines by immune cells, and endothelial function [13].

### 2.1. Plasminogen (Plg)

The inactive proenzyme Plg and its active derivative plasmin are key components of the fibrinolytic system. Plasmin belongs to the serine protease family of enzymes. Plg is secreted into the bloodstream mainly by hepatocytes and the kidney epithelium and can be synthesized and utilized out of circulation in the cornea [14]. Interleukin (IL)-1a and -1b can stimulate the extrahepatic synthesis of Plg in the human cornea [14].

Plg is a 92 kDa glycoprotein comprised of heavy (N-terminal domain followed by five kringle domains) and light (protease domain) chains linked by an activation loop [15]. Two glycosylated forms of Plg (I and II) vary in their number of sugar remnants, their affinity to fibrin, and their activation susceptibility [16].

Substrate or receptor binding leads to the dissociation of intramolecular bonds and proenzyme transition into an open form susceptible to activation. Partial autoproteolytic cleavage of 77 N-terminal amino acids by plasmin generates Lys-plasminogen, a transient form with increased affinity to fibrin and cell receptors [17]. Lys-plasminogen binding on monocyte/macrophage surfaces enhances their proteolytic potential [18]. Proteolytic fragmentation of the Plg/Pm molecule produces kringle-containing fragments such as angiostatins [19,20]. Angiostatins bind to plasminogen receptors or the hepatocyte growth factor receptor c-met. This binding results in the blockade of proliferation or angiogenesis and the induction of apoptosis [21].

α2-antiplasmin and α2-macroglobulin [22,23] are natural plasmin inhibitors (Figure 1). Proteolytically active plasmin has a broad specificity. It modulates the release and activation status of growth factors/cytokines (e.g., TGF-β, fibroblast growth factor-2 [24,25], hepatocyte growth factor [26], insulin-like growth factor-binding protein 4, and IL-1β [5]), hormones (e.g., prolactin [27], lactogen, osteocalcin [28], pro-opiomelanocortin [29], proinsulin [30,31]), receptors (e.g., uPAR [32] and EPH receptor A4), and proteases (e.g., tPA, uPA, and MMPs [33,34]) (Figure 1).

Plg and plasmin activators binding to annexin A2, urokinase plasminogen activator receptor (uPAR), and other docking sites colocalize enzyme and substrate, generating efficient plasmin at cell surfaces. Plg receptors can mediate the fibrinolytic function of this system and signal transmission inside the cell. Among the receptors for plasminogen on the cell surface are the highly specific Plg-RKT (plasminogen receptor with a C-terminal lysine) and the less specific αIIbβ3-integrin, αMβ2, αVβ3, α-enolase, gamma-actin, S100A10, annexin 2, histone H2B, amphoterin, or PAR. Aside from receptors, Plg interacts with partner proteins, such as fibrinogen/fibrin or tetranectin on cell surfaces [22,35]. Plg-RKT is expressed on monocytes, macrophages, and neuronal cells. It is sterically close to uPAR, providing conditions for plasmin generation and the Plg/Pm-dependent inflammatory response [36,37]. At the same time, annexin A2 reduces plasmin production and facilitates the autoproteolytic destruction of plasmin [38].

### 2.2. Plasminogen Activators (PAs)

tPA activates Plg mostly on fibrin thrombi surfaces but also on some cell membranes, mediating plasmin formation for cell movement through the extracellular matrix and modulating cell signaling. uPA mainly acts on cell surfaces. However, recent research has demonstrated the involvement of both PAs and plasmin in cell signaling, migration, and extracellular matrix remodeling [5,39]. Factor XIIa, an endogenous activator of the clotting system, can activate Plg and kallikrein and convert single-chain urokinase into double-chain urokinase. Nevertheless, their role in physiological fibrinolysis is considered insignificant [40].

tPA: tPA (tissue-type plasminogen activator, *Plat* gene) is a 70-kDa glycoprotein belonging to the serine protease family. tPA is synthesized mainly by endothelial cells, but mesenchymal cells, monocytes, smooth muscle cells, and fibroblasts can also produce it [41]. It is the primary PA (>90%) in all tissues except the kidney and liver (65%) and the spleen (40%). Lung tissues yield the highest tPA activity, followed by kidney, brain, heart, adrenal, liver, aorta, spleen, and muscle tissue [42]. In addition, stress, adrenergic stimulation, the diurnal cycle (and other circadian cycles), histamine, and thrombin enhance the synthesis and release of tPA. Most tPA in circulation exists in a complex with its primary inhibitor, PAI-1. tPA has a short half-life (3–4 min) and is removed from the bloodstream by the liver via mannose receptors [41,43]. The serine proteinase domain of tPA has a narrow specificity for Plg activation. In addition, the PAI-1 binding site is located in the serine proteinase domain of the tissue activator [44,45,46].

Besides having fibrinolytic functions, tPA can modulate cell signaling due to its ability to activate Plg on the cell surface or interact with specific receptors (reviewed in [8]). tPA-related signaling accelerates ischemic revascularization and regulates synaptic plasticity, blood-brain barrier permeability, cytokine production, cell proliferation, etc. tPA can affect cell fate alone or as part of the PAI-1/tPA complex [47]. The binding of tPA to Annexin A2 enhances proinflammatory cytokine production by macrophages through the generation of active plasmin and subsequent matrix metalloprotease-9 activation [48]. In addition, it stimulates endothelial progenitor cell evasion from the bone marrow [49].

The interaction of tPA with the low-density lipoprotein-related protein-1 (LRP1) receptor triggers cell survival and proliferation [39]. After binding to the membrane receptor LRP1, tPA-mediated NF-κB activation increases the expression of proinflammatory chemokines in macrophages [50]. tPA triggers a cascade of survival signaling involving extracellular signal-regulated kinase 1/2 [51]. In macrophages, enzymatically non-active tPA inhibits toll-like receptors through the N-methyl-D-aspartate receptor [52].

uPA: uPA (*Plau* gene) is a 54-kDa glycoprotein, synthesized as a single chain (sc-uPA) and converted into a two-chain uPA (tc-uPA) by plasmin and kallikrein [41]. The uPA molecule contains a protease, EGF, and a kringle domain without a lysine binding site which ensures that it cannot bind to fibrin [53].

Further proteolysis of sc- or tc-uPA by plasmin or matrix metalloproteases (MMPs) generates an amino-terminal fragment (ATF) that consists of the EGF and kringle domain (33 or 32 kDa) [54]. The ATF can bind to the primary uPA receptor called uPAR. Thrombin cleaves the Arg156–Phe157 peptide bond near the active site and generates another proteolytic two-chain inactive uPA [55]. Recent studies have demonstrated that soluble uPAR (suPAR) dimers, but not monomers, have a stronger binding ability to the ATF of uPA [56].

### 2.3. Plasminogen Activator Inhibitors

Fibrinolysis shutdown is provided by plasmin inhibitors, plasminogen activator inhibitors, and indirect fibrinolysis inhibitors. *α*2-antiplasmin and *α*2-macroglobulin [22,23] are naturally occurring specific plasmin inhibitors (Figure 1). Fibrin-bound plasmin and receptor-bound plasmin are protected from inactivation by plasmin inhibitors [7]. Indirect inhibitors (e.g., thrombin-activated fibrinolysis inhibitor, TAFI) regulate the rate of clot dissolution by fibrin modification [57]. However, the most abundant fibrinolysis inhibitor in circulation is PAI-1, a glycoprotein that belongs to serine protease inhibitors (SERPINs) and is, therefore, also called serpin E1. PAI-1 can be found in blood, soft tissues, tissues of the parenchymal organs, vessel walls, nervous tissue, etc. [58].

The primary function of PAI-1 in the bloodstream is to inhibit tPA and uPA, thus regulating the rate of fibrinolysis and the duration of blood clots. In tissues, PAI-1 also performs a signaling function and participates in the regulation of cell migration [59]. PAI-1 is synthesized in the liver and spleen epithelium, adipocytes, hepatocytes, platelets, megakaryocytes, macrophages, smooth muscle cells, and placental and endothelial cells [58]. In addition, pathological conditions can enhance PAI-1 expression in plasma (2–46 ng/mL) and other tissues. PAI-1 expression and release by cells such as platelets are regulated by various factors: growth factors (e.g., transforming growth factor-β (TGF-β), EGF, PDGF, tumor necrosis factor (TNF)-α, and interleukin-1 (IL-1)β), hormones (e.g., insulin, glucocorticoids, and angiotensin II), the glucosides and endotoxins of gram-negative bacteria, low-density lipoprotein, and very low-density lipoprotein [60].

PAI-1 is a single-chain glycoprotein with a mass of 47–50 kDa [61]. PAI-1 exists in several conformational forms. In addition to the active and inactive forms, there is also a latent form. PAI-1, synthesized as an active enzyme, is spontaneously transformed into an inactive form with a half-life of 1 to 2 h. PAI-1 is the only serpin that can perform a reverse conformational transition between active and latent states. In plasma, the active form of PAI-1 can be stabilized by binding to vitronectin, thereby increasing its half-life in the bloodstream. On the other hand, PAI-1 activity rapidly decreases at lower pH, as has been found in ischemic tissues [59].

## 3. Soluble uPAR, a New Biomarker of Inflammation

uPAR (CD87; plasminogen activator, urokinase receptor (*Plaur* gene)) is a membrane-linked protein found in immunologically active cells (monocytes, neutrophils, activated T lymphocytes, macrophages), endothelial cells, keratinocytes, fibroblasts, smooth muscle cells, megakaryocytes, and certain tumor cells. uPAR can interact with uPA, integrin, or other partners, including vitronectin, high molecular weight kininogen, G protein-coupled receptors, and tyrosine kinase receptors that can trigger plasmin generation and degradation of the extracellular matrix (ECM) along the leading edge of a migratory cell [62] and activate downstream signaling pathways. uPA can increase cell proliferation via plasmin through the proteolytic activation of growth factors and adhesion molecules. It can also remodel tissues or the ECM and regulate the adhesion and invasion of normal cells and cancer cells [54].

During inflammation, microbe-mediated toll-like receptors (TLRs) or cytokine receptor stimulation upregulate uPAR expression in immune cells through the binding of transcription factors such as the nuclear factor kappa-light-chain-enhancer of activated B cells and activator protein 1 to the promoter region of the PLAUR gene (reviewed in [63]). uPAR is expressed at the cell surface and bound to the membrane via a glycosyl phosphatidylinositol (GPI) anchor. The membrane-bound form of uPAR is not covered in this review (see [64]), but it has been explored as a drug target, e.g., in cancer treatments.

Cleavage of uPAR at the GPI anchor by proteases such as plasmin can shed the extracellular part of uPAR, releasing the soluble form of the receptor (suPAR) into the blood, mucosa, urine, and saliva [65]. SuPAR consists of three domains (D1, D2, and D3) that retain most of the uPAR activities, namely cellular attachment, motility, and migration through its interaction with integrins. Cleavage of uPAR/suPAR in the linker between D1 and D2 by uPA and MMPs generates D1 and D2D3 fragments [66]. These uPAR cleavage products serve different functions. The full-length uPAR and suPAR (but not D1 or D2D3 fragments) engage with uPA and promote ECM degradation [67]. In contrast, only the D2D3 fragment binds to the chemotaxis-mediating formyl peptide receptors and supports the inflammatory response [68]. Cytokines that can cause inflammation or increase blood leukocytes, such as the cytokine granulocyte-stimulating factor, increase circulating suPAR and D1 or D2D3 fragments [34,69]. In cells where uPA–uPAR is active, full-length suPAR acts to downregulate promigratory signaling, probably by competitive displacement of the uPA–uPAR complex from signaling adaptor proteins [70]. The proteolytic cleavage of suPAR to produce the D2D3 form increases its signaling activity, likely because the cleaved form has a similar signaling ability to that of uPA.

suPAR is removed from circulation by the kidney [71]. Therefore, patients suffering from renal diseases such as focal segmental glomerulosclerosis show elevated blood suPAR levels. A causal role of suPAR has been described in chronic kidney disease. High circulating suPAR levels have induced renal injury in experimental models and suPAR infusion-induced proteinuria in uPAR-knockout mice [72]. suPAR, rather than C-reactive protein (CRP) has been proposed as a biomarker for bacterial infection. suPAR, not CRP, carries the strongest predictive value of the three inflammatory biomarkers (CRP, procalcitonin, and suPAR) in sepsis patients. suPAR has diagnostic value [73]: suPAR concentrations of >12.9 ng/mL measured within the first 24 h of diagnosis predicted death in the first 28 days in a group of 180 hospitalized Greek patients in the intensive care unit.

## 4. Fibrinolytic Factors during Inflammation in the Oral Cavity

Oral mucosa covers the oral cavity, which is lined with the keratinized and non-keratinized stratified squamous epithelium. It is moistened with excretions of the major parotid, submandibular, sublingual, and minor salivary glands within the oral cavity. Mechanical mucosal trauma occurs while eating, drinking, and talking (and even from tobacco inhalation). The oral mucosa is a first-line defense that interacts with pathogens (e.g., bacteria, viruses, or fungi) and provides specific (immune) or non-specific protective responses against pathogenic microorganisms via pattern recognition receptors including C-type lectin receptors (Dectin-1, Dectin-2) or TLR1-1. The gradual desquamation of the mucosal epithelium is a protective mechanism which eliminates adherent pathogenic microorganisms and prevents their further invasion of underlying tissues [74]. A host organism reacts with the release of proinflammatory cytokines and proteases to fight oral microorganisms in the gingiva and periodontal ligament space.

Salivary glands provide local mucosal specific and non-specific immunity. The proper qualitative and quantitative composition of saliva and salivation rate protect and maintain the integrity of the oral cavity. Salivary mucins avert plaque formation on teeth surfaces via bacteria binding. Salivary lysozyme, an enzyme that lyses bacteria cell walls, prevents the overgrowth of oral microbiota.

Several studies are available regarding the fibrinolytic properties of salivary glands and other fluids of the oral cavity [75] during steady state and stress/inflammation. Salivary suPAR, tumor necrosis factor α (TNF α), and interleukin (e.g., IL-1β) levels increased in healthy subjects exposed to psychological stress [76] and showed a strong positive baseline and post-stress correlations. Gingivitis occurs due to bacteria accumulation in plaques on the cervical margins of teeth. Thus, children with gingivitis, but not those in the healthy control group, had higher salivary suPAR levels, and this correlates with gingivitis disease severity [77]. Elevated saliva suPAR levels have been detected and proposed as a biomarker of gingivitis and periodontitis [78,79]. In addition, salivary suPAR level indices, such as the gingival index and simple oral hygiene index, can indicate periodontal conditions. Therefore, suPAR levels in saliva can mirror the systemic inflammatory or stress response.

suPAR levels are high in saliva and do not correlate with plasma suPAR levels. This suPAR expression pattern is surprising given that biomarkers are usually lower in saliva compared with plasma/serum [80]. One reasonable explanation for the lower detection of suPAR in plasma when compared with saliva is that the inflammatory response in gingiva is not strong enough to produce a systemic response. Aside from suPAR, Plg receptors such as glyceraldehyde-3-phosphate dehydrogenase, α-enolase, and annexin A2 are also found in the saliva [81,82,83,84], but their function in the oral cavity remains unclear.

**Figure 2:**
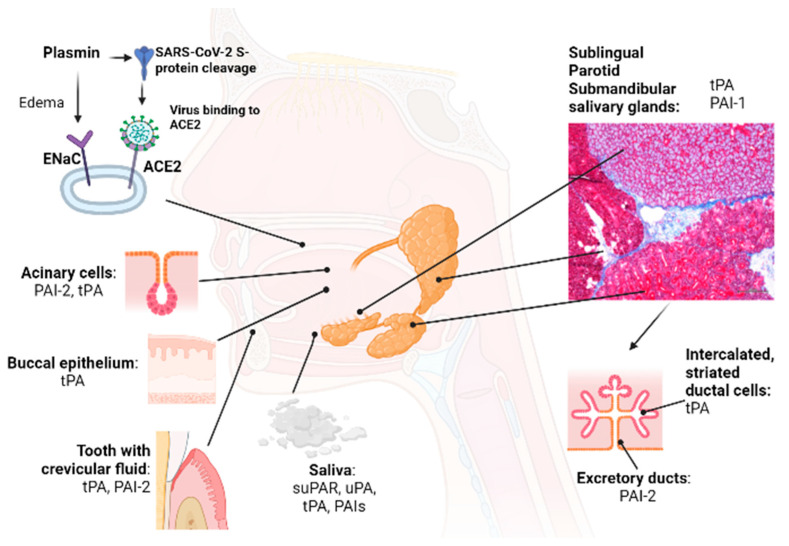
**Organs/tissues that contribute to the plasminogen/plasmin system in the oral cavity.** Major and minor salivary glands, oral mucosae, and the periodontium play an important role in maintaining the Plg/Pm system balance in the oral cavity. Oral cavity chronic diseases and hyposalivation cause an imbalance in the Plg/Pm system in the oral cavity, supporting inflammation. Viruses, including SARS-CoV2, enter the body via the oral and nasal cavities, where the initial replication of viruses occurs. The non-fibrinolytic proteolytic function of plasmin supports the initial stages of infection with SARS-CoV-2. For example, the SARS-CoV-2 virus can enter the buccal epithelium after binding its S protein to ACE2. Plasmin cleaves the S protein of the SARS-CoV-2 virus and ENaC α, β, γ and facilitates SARS-CoV-2 infection of susceptible cells (non-fibrinolytic function). Abbreviations: tPA, tissue-type plasminogen activator; PAI-1,2, plasminogen activator inhibitor-1,2; Plg, plasminogen; uPA, urokinase-type plasminogen activator; ACE2, angiotensin-converting enzyme 2; ENaC, epithelial sodium channel.

The functional salivary gland unit consists of intercalated and striated ductal cells. Excretory ducts and acinar cells highly express PAI-2. tPA is expressed in serous but not mucous acini [75]. Overall, several independent studies have confirmed the existence of tPA in saliva, but the results of different investigators varied concerning the presence/dominance of PAI-1 or PAI-2 in salivary gland tissues. In the late 1980s, physiologically active tPA (but not uPA or Factor XII) was identified in human unstimulated saliva derived from the submandibular/parotid glands or buccal epithelial cells of healthy volunteers. In these early studies, active fibrinolytic tPA and uPA in saliva were identified using fibrin plates containing Plg and specific antibodies against tPA or uPA. Buccal-epithelial cells produce tPA, while the activity of tPA in parotid and submandibular saliva is low [85,86] (Figure 2). In another study, tPA, PAI-1, and PAI-2 were expressed in salivary tissues, but PAI-2 was reported as the primary PA inhibitor in saliva, especially in males [75]. In follow-up studies, PAI-1 was detected in saliva and did not show daytime differences [87]. These conflicting results concerning the presence or absence of PAI-1 in saliva might be due to differences in the available methods during different periods.

PAI-1 increases in saliva were associated with insulin resistance and inflammation and regarded as a proinflammatory marker and valuable diagnostic marker to track periodontal therapy [88]. Gingival crevicular fluid is an inflammatory exudate from the periodontal tissues composed of serum and locally generated materials such as tissue breakdown materials, inflammatory mediators, and antibodies directed against dental plaque bacteria (Figure 2). It plays a specific role in maintaining the structure of junctional epithelium and the antimicrobial defense of the periodontium. High tPA and PAI-2 levels that normalized after periodontal therapy were found in the crevicular fluid of patients with inflammatory periodontal diseases. Gingival crevicular fluid volume positively correlates with clinical parameters, especially gingival bleeding. In multivariate regression models, higher CRP and tPA levels correlated with self-reported periodontal disorders [89].

### Plg Deficiency and the Oral Cavity

Plg deficiency due to mutations in the *Plg* gene results in changes in its function and causes extensive extracellular matrix deposition, impaired fibrin clearance, excessive neutrophil activation, or fibrosis [90,91,92]. Moreover, patients with congenital Plg deficiency first manifest symptoms of disease in the oral cavity. There are two types of Plg deficiency: hypoplasminogenemia (type I Plg deficiency), in which the level and activity of Plg are reduced, and dysplasminogenemia (type II Plg deficiency), in which the level of immunoreactive Plg is within the normal range, but the specific activity of Plg is reduced. Type 1 Plg deficiency is a rare autosomal recessive disease caused by homozygote or compound-heterozygote mutations of the Plg 6q26 gene, and it has an incidence of 1.6 in 1 million individuals (reviewed in [93]). Plg type 1 deficiency causes recurrent, wood-like (ligneous) pseudomembrane formation on mucosal surfaces. Patients suffer from severe mucosal inflammatory diseases with ligneous periodontitis and other manifestations in the eye (ligneous conjunctivitis [93] is the most common clinical observation), lung, vagina, and gastrointestinal tract [94,95]. Periodontitis is usually accompanied by severe gingiva hypertrophy and rapid alveolar bone destruction that leads to tooth loss and abnormal mucosal wound healing [96]. However, clinical symptoms were observed to disappear in Plg-deficient patients following replacement therapy with Plg [97].

Plg enhances the clearance of fibrin, preventing the excessive build-up of fibrin fibers [94,98]. Commensal microorganisms trigger the deposition of fibrin in oral mucosae. Silva et al. reported that, like humans, mice lacking Plg accumulate extravascular fibrin and develop an oral pathology that phenocopies human ligneous periodontitis [90]. Excess fibrin can activate neutrophils through binding via the αMβ2 integrin receptor, produce reactive oxygen species, and form neutrophil extracellular traps (NETs) [90]. NETs contain histones as well as granular enzymes and peptides, including neutrophil elastase, myeloperoxidase, cathepsin G, leukocyte proteinase 3, lactoferrin, gelatinases, lysozyme C, calprotectin, neutrophil defensins, and cathelicidins [99]. NETs form a finer structure within the pores of the larger fibrin structure to prevent pathogen escape. The inclusion of fibrin strengthens NETs and suppresses their degradation by plasmin [100]. Ultimately, plasmin will remove these NETs. While NETs can promote the fight against bacteria in the oral cavity, neutrophils within NETs damage the periodontal tissues [90].

During chronic periodontitis, periodontal bacterial pathogens create a pro-fibrinolytic environment in the place of tissue invasion. A haplotype block downstream of Plg (rs1247559) is associated with chronic and aggressive periodontitis in German subjects [101]. The periodontal pathogen *Tannerella forsythia* produces the serpin-type plasmin inhibitor miropin, a protease inhibitor of the serpin superfamily. Miropin is a specific and efficient plasmin inhibitor [102]. Plasmin activity in crevicular fluid decreases after periodontal treatment in patients with diverse inflammatory periodontal diseases.

## 5. Fibrinolytic Factors during Bacterial and Viral Infection

Inflammation is the immune system’s response to microbes or toxic agents (chemicals, irradiation) and damaged cells, and its aim is to remove harmful stimuli and initiate healing. Plg and fibrinogen modify the inflammatory response in vivo and contribute to bacterial virulence and host defense. The role of fibrinolytic factors in the inflammatory response process has been reviewed elsewhere [8,9]. Infectious pathogens utilize the Plg/Pm system for host ECM degradation and tissue invasion. As a rapidly assembled provisional matrix protein, fibrin(ogen) is an early line of host defense which limits bacterial growth, suppresses microbe dissemination, and mediates host bacterial killing. Bacteria, pathogenic fungi, and parasitic protists express Plg receptors on their surface to convert Plg into plasmin vis host tPA/uPA or express bacterial PAs such as streptokinase or staphylokinase [103,104,105]. Bacterial factors can bind fibrinogen or fibrin, promote fibrin polymer formation, or dissolve fibrin. The involvement of fibrinolytic factors in the pathogenesis of bacteria has been extensively studied and will not be reviewed here [106,107,108]. Here, we provide some examples of the mode of action of fibrinolytic factors: pathogenic bacteria use bacterial Pas, such as streptokinase and staphylokinase, and receptors for host tissue invasion [105]. Infectious bacteria such as Streptococcus, Hemophilus, and Neisseria enter mammalian cells via the Plg receptor α-Enolase [109].

Furthermore, bacteria use Plg/plasmin to evade the complement cascade: co-binding with Plg to the bacterial membrane enhances the activity of the C4-binding protein, the inhibitor of C3 convertase [110]. Another example is plasmin, which cleaves C3 and C5 complement components [111]. In other words, microorganism-infected cells alter the “proteolytic, fibrinolytic niche” (proteases including fibrinolytic factor in a narrow space) or modulate fibrinolytic receptor expression on mammalian cells to promote their propagation in host cells.

Vascular endothelial damage is a critical step in the pathophysiology of organ damage after bacteria or virus infection. Vasculature-lining endothelial cells are bioreactors that produce or contribute to the modulation status of cytokines, coagulation, and fibrinolytic system factors. An overproduction or imbalance of fibrinolytic/coagulation factors and inflammatory cytokines can contribute to clinically severe cases of inflammation, referred to as cytokine storm syndrome. We demonstrated that increased circulating plasmin levels can result from severe inflammation during graft-versus-host disease and lipopolysaccharide stimulation causing septic shock, and we also observed increased circulating plasmin levels in a model of macrophage activation syndrome established through the activation of TLR-9 [112,113,114]. Plasmin blockade prevented the deadly cytokine storm syndrome in these murine models of severe inflammation partly through its ability to suppress MMP and proinflammatory cytokine release.

Researchers have identified the “cytokine storm” as an inflammatory cytodynamic control mechanism that contributes to the aggravated pathology of coronavirus disease 2019 (COVID-19). COVID-19 is a highly contagious infectious disease caused by the severe acute respiratory syndrome coronavirus 2 (SARS-CoV-2) virus. It is now widely accepted that the fibrinolytic system is implicated in the initial stage of infection and invasion by SARS-CoV-2, the virus causing COVID-19. SARS-CoV-2’s major entrance routes are the oral and nasal cavities. In addition, the Plg/Pm system is a significant player in the inflammatory response and contributes to disease severity (reviewed in [115]).

### 5.1. Viral Entry of SARS-CoV-2 in the Oral Cavity

Viruses such as the human papillomavirus and SARS-CoV-2 can enter the body through the oral cavity. Earlier studies have demonstrated that Plg enhances viral inflammation caused by the influenza viruses H5N1 and H1N1 in mice [116]. In addition, Plg and plasmin levels increase during viral infection in murine lungs, causing enhanced fibrinolysis that ultimately results in the occurrence of FDP and D-dimers, degradation products of fibrinolysis.

The oral and nasal cavities, with saliva as a viral carrier, are exploited by SARS-CoV-2 [117]. The virus expands in the oral mucosa, periodontal tissues, salivary glands, tongue, nasal cavity tissues, and olfactory bulb tissues [118]. The saliva contains large amounts of SARS-CoV-2 secreted from salivary gland epithelial cells. When infected saliva is swallowed, or tiny particles of it are inhaled, the virus can transmit further into our throats, lungs, or guts. These data indicate that understanding virus infection and replication in the oral cavity is vital in determining systemic infection.

The infection of cells requires cleavage at the S1/S2 and the S2` sites of spike proteins. Spike (S) proteins assemble into trimers on the SARS-CoV-2 virion surface, forming a “corona”, or crown-like, appearance. One S complex consists of two N-terminal domains (“S1”), responsible for receptor binding, and a C-terminal S2 domain that is required for cell fusion [119]. Salivary gland cells, namely ductal epithelium and serous acinar cells, highly express the angiotensin-converting enzyme 2 (ACE2) receptor [120] and TMPRSS receptors (Figure 2) [121,122].

The S1 subunit of the S protein engages ACE2, and viral entry into the host cell is facilitated. The viral particle incorporates the S protein, which has already undergone S1/S2 cleavage by furin protease [123]. It then undergoes further cleavage at the S2′ site, mediated by the type II transmembrane serine protease 2 (TMPRSS2), after binding to ACE2 to facilitate membrane fusion at the plasma membrane (reviewed in [124]). The S1/S2 cleavage can also be performed by plasmin and trypsin [125]. Following its attachment to the spike protein, ACE2 is internalized and downregulated [115].

TMPRSS2 is a crucial protease for S protein activation, which leads to the interaction of viral fusion proteins with host cell receptors. Aside from SARS-Co-V-1 and -2, S protein activation can occur in Middle East respiratory syndrome and influenza A and B via TMPRSS2 [126,127]. Inhibition of the proteolytic activity of TMPRSS2 blocks viral particle maturation and cell invasion, thus lowering viral load [128]. Earlier studies have demonstrated that PAI-1 is a physiological TMPRSS-2 inhibitor [129]. Plasmin has been shown to cleave the S protein of SARS-CoV-2 in an in vitro system involving luciferase activity of SARS-CoV-2 pseudovirus-infected HEK293 cells stably expressing human ACE2 [130].

Salivary glands also express epithelial Na^+^ channel (ENaC) α, β, γ [120], especially during inflammation, infection, or trauma. ENaC α, β, γ proteins are localized in ductal epithelial cells, and the highest amounts are found in serous acinus cells of the parotid and submandibular glands [121]. Plasmin can cleave ENaC γ and may play a role in edema development during disease progression [131].

Povidone iodide, a well-known antiseptic, can inhibit plasmin, and possibly MMPs, in the oral cavity, which might explain its efficacy against COVID-19 infection [132]. Recent research has confirmed that the risk of several adverse events such as ICU admission, death, and the need for mechanical ventilation in patients with COVID-19 is considerably higher in patients with periodontitis than patients with a healthy periodontium [133]. Therefore, it will be interesting to determine the role of plasmin in these patients.

### 5.2. Coagulopathy with Hyper-/Hypocoagulation during COVID-19

Hypercoagulation is commonly found in COVID-19 patients. The important roles of plasmin and the fibrinolytic system in counterbalancing thrombotic events occurring during COVID-19 are not the subject of this review, but they have been reviewed elsewhere [8,134,135]. Biomarkers (reviewed in [136]), including those of the fibrinolytic system (fibrinogen, Plg, fibrin, D-dimers, suPAR, tPA, and PAI-1), can predict COVID-19 severity or mortality [137,138] (reviewed in [136]). A state of hyperfibrinolysis, characterized by increased fibrin degradation products (such as D-dimers) and reduced platelets, is associated with high mortality rates in COVID-19 patients [139]. In addition, low Plg levels predicted mortality in a group of COVID-19 patients from Italy [140].

Race and ethnicity are risk factors that can affect socioeconomic status, determining access to health care and, consequently, health after SARS-CoV-2 infection [141]. Following adjustment for age, body mass index, and history of cardiovascular diseases, we found that German patients exhibited a hyperactive inflammatory response and coagulopathy with hypercoagulation, a pattern reported for Western countries. In contrast, Japanese patients in the same clinical phase of the disease presented with a suppressed inflammatory response and coagulopathy with hypocoagulation [141]. There might be several explanations for the lower disease activity as determined by the inflammatory and coagulation/fibrinolysis response: the dedication of Japanese citizens to consistently wearing masks in public spaces to prevent oral dissemination of the virus, other socioecological factors, or other yet-to-be-determined genetic differences in critical genes in both populations.

## 6. Concluding Remarks and Future Directions

The oral cavity, with all of its components, is a complex of organs that are a first line defense against most viral and bacterial pathogens. The fibrinolytic factors of the Plg/Pm system, their soluble and membrane receptors, and fragments such as suPAR modulate physiological and pathological conditions, especially inflammation. Some of these molecules have signaling functions. The biological functions of full-length proteins and their fragments can be anticipated. This review aims to highlight the functions of the Plg/Pm system in the oral cavity. Under physiological conditions, fibrinolytic factors are present in the oral cavity and are secreted mostly with saliva. However, plasmin, and therefore fibrinolysis, is not activated. This changes during inflammation. Viruses such as SARS-CoV-2 exploit the fibrinolytic system to promote host cell infection. Fibrinolysis, the removal of fibrin, is the primary function of fibrinolytic factors. However, non-fibrinolytic functions, such as the cleavage properties of plasmin, come in handy here: cytokines or proteases (MMPs) are activated and receptors such as suPAR are shed from the surface promoting cell migration and modulation of the inflammatory response.

Several investigators have confirmed the expression of fibrinolytic factors in the oral cavity. However, future studies will be necessary to establish the functions of the Plg/Pm system under physiological and pathological conditions in the oral cavity, including the salivary component. Understanding the mechanism and pathophysiology underlying the Plg/Pm system in the oral cavity will enable us to identify novel treatment targets that can ultimately reduce or prevent microbe-driven diseases.

## Data Availability

No data are presented. Figures are original.

## Abbreviations

ACE 2, angiotensin-converting enzyme 2; ATF, amino-terminal fragment; COVID-19, coronavirus disease 2019; EGF, epidermal growth factor; ECM, extracellular matrix; ENaC, epithelial Na^+^ channel; GPI, glycosyl phosphatidylinositol; ICU, intensive care unit; IL-1, interleukin-1; LRP1, low-density lipoprotein-related protein-1; MMPs, matrix metalloproteinases; NETs, neutrophil extracellular traps; NMDAR, N-methyl-D-aspartate receptor; PA, plasminogen activator; PAI-1, inhibitor of plasminogen activator 1; PAI-2, inhibitor of plasminogen activator 2; PAR, protease-activated receptor; PDGF, platelet-derived growth factor; uPAR, urokinase-type plasminogen activator receptor; suPAR, soluble urokinase plasminogen activator receptor; Plg-RKT, plasminogen receptor with a C-terminal lysine; PLAUR, plasminogen activator, urokinase receptor; Pm, plasmin; TGF-β, transforming growth factor beta; tPA, tissue-type plasminogen activator; uPA, urokinase-type plasminogen activator; SERPINs, serine protease inhibitors, SARS-CoV-2, severe acute respiratory syndrome coronavirus 2; TLRs, toll-like receptors; TMPRSS, transmembrane protease serine.
